# Modifying the thermal conductivity of small molecule organic semiconductor thin films with metal nanoparticles

**DOI:** 10.1038/srep16095

**Published:** 2015-11-04

**Authors:** Xinyu Wang, Kevin D. Parrish, Jonathan A. Malen, Paddy K. L. Chan

**Affiliations:** 1 Department of Mechanical Engineering, The University of Hong Kong, Hong Kong; 2 Department of Mechanical Engineering, Carnegie Mellon University, Pittsburgh, Pennsylvania, USA

## Abstract

Thermal properties of organic semiconductors play a significant role in the performance and lifetime of organic electronic devices, especially for scaled-up large area applications. Here we employ silver nanoparticles (Ag NPs) to modify the thermal conductivity of the small molecule organic semiconductor, dinaphtho[2,3-b:2’,3’-f]thieno[3,2-b]thiophene (DNTT). The differential 3-ω method was used to measure the thermal conductivity of Ag-DNTT hybrid thin films. We find that the thermal conductivity of pure DNTT thin films do not vary with the deposition temperature over a range spanning 24 °C to 80 °C. The thermal conductivity of the Ag-DNTT hybrid thin film initially decreases and then increases when the Ag volume fraction increases from 0% to 32%. By applying the effective medium approximation to fit the experimental results of thermal conductivity, the extracted thermal boundary resistance of the Ag-DNTT interface is 1.14 ± 0.98 × 10^−7^ m^2^-K/W. Finite element simulations of thermal conductivity for realistic film morphologies show good agreement with experimental results and effective medium approximations.

With excellent mechanical flexibility, low fabrication cost, and compatibility with large area fabrication, organic semiconductors have become a promising candidate as the next generation of flexible electronics^1^,^2^,^3^,^4^,^5^,^6^,^7^,^8^. The rapid development of organic semiconductors, including the synthesis of new materials and the processing of active layers, provides optimal packing and thus higher carrier mobility in organic thin films or single crystal materials^7^,^8^. For example, the polycrystal and single crystal forms of 2,9-didecyl-dinaphtho[2,3-b:2′,3′-f]thieno[3,2-b]thiophene (C10-DNTT) have shown a carrier mobility of 8.5 cm^2^/V-s^9^ and and 11 cm^2^/V-s^10^, respectively. Increasing carrier mobility will yield larger currents as well as parasitic heating during operation, especially for large area electronics where the device density is high. Generally, the thermal conductivity of organic semiconductors are much lower than traditional inorganic semiconductors and typically have a value below 1 W/(m-K)^11^,^12^,^13^,^14^,^15^,^16^.

The inferior thermal transport properties and susceptibility to high temperatures of organic semiconductors often lead to the degradation of the organic device performance^4^,^17^,^18^. Chung *et al.* demonstrated that the increase of operating temperature of organic light emitting diodes (OLED) would reduce the lifetime^4^. Moreover, it has also been reported that the carrier mobility of planar pentacene field effect transistors decreased one order of magnitude after 20-min exposure to 60 °C under vacuum^17^. For electrically conductive polymers, thermal induced degradation is also equally important. For example, in poly(3,4-ethylenedioxythiophene): poly(styrenesulphonate) (PEDOT:PSS), it has been shown that continuous heating of the material at 120 °C will cause shrinkage of the PEDOT conductive grains and thus decrease the electrical conductivity of the film^18^. Hence, the capability of organic semiconductors to effectively transport and dissipate heat is critical to the overall performance and lifetime of next generation organic electronics.

Recently, experiments and simulations have been developed to study the thermal conductivity of various organic semiconductors used in solar cells or transistors, such as PCBM^11^,^19^, P3HT^11^, C_60_^16^, and pentacene^12^. These organic semiconductors can be classified as either polymers or small molecules. The former usually have a high sublimation temperature due to their long chains, and thus are mainly prepared by a solution-process method, such as spin-coating. The latter have a lower sublimation temperature, and hence are typically deposited by thermal evaporation under a vacuum environment. Since small molecules are held together by weak van der Waals forces, the quality of small molecule organic thin films formed by thermal evaporation depend strongly on the deposition conditions, such as the growth rate, deposition temperature, or impurity concentration. Weak intermolecular interactions of organic semiconductors suggest the potential to adjust the thermal conductivity of organic thin films, an area which has not been extensively investigated.

Although the modification of the thermal conductivity of organic thin films has not been extensively studied, new work focused on organic thermoelectric materials requires insight to their thermal properties. Recently, Kim *et al*. reported that a thermoelectric figure of merit (ZT) of about 0.42 could be achieved for organic semiconductors by properly doping poly(styrenesulphonate) into poly(3,4-ethylenedioxythiophene) (PEDOT:PSS) with dimethyl-sulphoxide (DMSO)^20^. With a particular focus on modifying the power factor, tellurium nanowires have also been added into PEDOT:PSS to enhance the power factor by five orders of magnitude in work by Yee *et al*.^21^. It is however important to point out that most of these organic thermoelectric materials with a reportedly high ZT or power factor are prepared with a polymer based PEDOT:PSS, and thermoelectric studies of small molecule organic semiconductors are very limited. To explore this area our group has recently demonstrated that the electrical conductivity of pentacene thin films can be enhanced by sandwiching a thin layer of silver nanoparticles (Ag NPs) between two pentacene layers. This metal-organic hybrid material can be used as the active layer for thermistors and memory transistors^2^,^22^,^23^. In support of this approach, Jin *et al*. embedded Ag NPs into thin copper phthalocyanine (CuPc) films and observed a four-order of magnitude increase in the electrical conductivity for Ag volume fractions exceeding 30%^13^. They also demonstrated that the thermal conductivity showed a decreasing trend when the Ag volume fraction was less than 10% and increased when the Ag volume fraction was higher than 10%. All of these findings suggest that embedding metal nanoparticles into small molecule organic materials is an effective method to adjust the electrical and thermal properties of metal-organic hybrid materials.

In the current work, our focus is to utilize the differential 3-ω method^24^,^25^ to study the thermal properties of dinaphtho[2,3-b:2′,3′-f]thieno[3,2-b]thiophene (DNTT), which is a novel thienoacene-based organic semiconductor for transistor devices. The sulfur atoms in the DNTT molecules are integrated into hydrocarbons with an acene structure to enhance their stability under ambient air conditions^26^,^27^,^28^,^29^. The 3-ω method has been widely used to measure the thermal conductivity of thin films^24^. As shown in Fig. 1(a–e), a metal wire deposited onto the sample performs as a heater and thermometer at the same time. When the metal wire is driven by an AC current, it periodically heats the sample and its resultant temperature change can be monitored by its resistance. According to the temperature change of thin film sample and reference sample, the thermal conductivity of the thin film can be extracted^25^. (see Method for details)

We demonstrate that the modification of the cross-plane thermal conductivity (*k*_*film*_) of DNTT thin films can be achieved by employing different volume fractions of Ag NPs. We also investigate the correlation between the *k*_*film*_ and the deposition temperature of the DNTT thin films. We observe that even though the DNTT thin films deposited at an elevated temperature increasing from 24 °C to 80 °C have a larger grain size, the effects of the deposition temperature and subsequent morphological changes on the thermal conductivity are very limited. On the other hand, we observe that the *k*_*film*_ of Ag-DNTT hybrid thin films shows a trend of an initial decrease when the Ag volume fraction increases from 0% to 16% then *k*_*film*_ increases afterwards to the maximum studied Ag volume fraction of 32%. Then we apply the effective medium approximation (EMA) to theoretically calculate the thermal conductivity of the hybrid thin films. By comparing the calculated results with the experimental results of thermal conductivity, we are able to further extract the thermal boundary resistance (TBR) of the Ag-DNTT interface. Finally, the finite element method (FEM) is applied to simulate the thermal conductivity of hybrid thin films with realistic morphologies and finds good agreement with experimental results. Consequently, the results of this work will provide useful information for engineering the thermal conductivity of small molecule organic thin films and thermal management in organic electronics.

## Results

### Effect of deposition temperature

The AFM images of the DNTT thin films deposited at different substrate temperatures, ranging from 24 °C to 80 °C, are shown in Fig. 2(a). The grain size of the DNTT clearly increases with deposition temperature. The sample deposited at 80 °C, demonstrates three-fold increase in the average grain size, from 126 nm to 451 nm, relative to the sample deposited at 24 °C. The corresponding *k*_*film*_ of the DNTT thin films at different deposition temperatures is shown in Fig. 2(b). Even though the grain size increases at higher deposition temperatures, *k*_*film*_ is relatively constant. The values are 0.45 ± 0.06 W/m-K at 24 °C, 0.47 ± 0.08 W/m-K at 40 °C, 0.43 ± 0.07 W/m-K at 60 °C and 0.41 ± 0.07 W/m-K at 80 °C. The invariance of *k*_*film*_ suggests that the density of the grain boundaries in the in-plane direction does not affect the vertical heat conduction path.

To verify the cross-plane crystallinity of the DNTT deposited at different temperatures, we utilized the XRD to measure the out-of-plane crystal structure of the DNTT thin films. From the out-of-plane XRD spectra shown in Fig. 2(c), we can see that there are three diffraction peaks, (001), (002) and (003). The (001) peak at around 2θ = 5.57° shows the highest intensity, thus suggesting that the d-spacing (between lattice planes parallel to the substrate) in the z-axis direction is 1.59 nm, which is similar to previously reported values^28^,^30^. The values of the 2θ diffraction peaks are the same for DNTT films deposited at different temperatures, which indicates that the d-spacings in the z-axis direction are identical among these films. It is noteworthy to point out that although grain size may affect the in-plane thermal conductivity, its impact on heat dissipation in thin film electronic devices is limited. Herein we are focusing on modulation of the cross-plane thermal conductivity.

### Effect of Ag NPs

We modified *k*_*film*_ of the DNTT thin films by adding extra boundaries or interfaces along the heat conduction path in the cross-plane direction. Here, we applied a thin layer of Ag NPs onto the DNTT thin films, and the measured *k*_*film*_ as a function of the Ag volume fraction (0% to 32%, proportional to the effective thickness of the deposited Ag) is shown in Fig. 3. Both the DNTT and Ag NPs were deposited onto the substrate which was maintained at room temperature. As shown in Fig. 3, *k*_*film*_ initially decreases to an Ag volume fraction of 16% and then gradually increases when the Ag volume fraction is further increased. At an Ag volume fraction of 16%, *k*_*film*_ reaches a minimum value of 0.27 ± 0.04 W/m-K, which represents a 40% decrease in comparison with the pure DNTT thin film. A similar effect was observed by Jin *et al*. under copper(II) phthalocyanine (CuPc) and silver^13^. Here we hypothesize that this reduction in thermal conductivity is mainly due to two reasons: (i) large thermal boundary resistance between the DNTT and the Ag NPs that occludes their contribution to thermal conductivity; (ii) crystallinity modification of DNTT by Ag NPs which we will discuss later. When the Ag volume fraction is greater than 28%, the benefit of the Ag’s superior thermal conductivity begins to outweigh the effects of the thermal boundary resistance and crystallinity modification, and thus *k*_*film*_ has a higher value than that of the pure DNTT thin films.

Figure 4(a) shows top view SEM images of the Ag NP layer deposited onto the top surface of the bottom DNTT film. These images demonstrate that when the Ag volume fraction is small, the Ag is in NP form, and the NPs gradually grow into islands as the Ag volume fraction increases. For the sample with an Ag volume fraction of 32%, small islands aggregate together to form larger islands and the average distance between the islands is reduced to approximately 10 nm. From the cross-sectional TEM images in Fig. 4(b), we observe that despite using the layer-by-layer method to deposit the Ag and DNTT, the Ag NPs penetrate into the bottom DNTT layer and form a discontinuous phase in the nanocomposite structure. When the Ag volume fraction is 32%, the Ag layer forms into a nearly continuous layer, which is consistent with our observations of the top view SEM images. Note that the Ag layer below epoxy in TEM images is intentionally deposited to distinguish the epoxy from the DNTT layer in TEM samples, and is not included in thermal conductivity measurement samples.

When the Ag is in a bulk form, the electrons mainly contribute to the thermal conductivity and their electrical and thermal conductivities are correlated by the Wiedemann-Franz law. When the Ag NP size is small and comparable to the electron mean free path^31^, the electron transport will become ballistic and boundary scattering will play a predominant role. As a result, the effective electrical and thermal conductivities will significantly decrease in comparison to the bulk values^31^,^32^. We attribute the observed trend of decrease in the *k*_*film*_ when the Ag volume fraction is less than 16% to the combined low thermal conductivity of the Ag NPs in the nano-island form, crystallinity modification of DNTT by Ag NPs, and the thermal boundary resistance induced at the Ag-DNTT interface. Scattering of heat carriers at this interface hinders the energy transport between the two dissimilar materials, limits thermal current though the Ag, and ultimately reduces the thermal conductivity of the hybrid thin films. Despite a certain degree of thermal annealing on the DNTT films that may take place during the deposition of the Ag NPs, the previously observed constant *k*_*film*_ at different deposition temperatures and the non-monotonic trend with Ag concentration suggest that thermal annealing is not the primary source of variation in the thermal conductivity shown in Fig. 3.

Figure 5(a) shows the out-of-plane XRD spectra of Ag-DNTT hybrid thin films with Ag volume fractions of 0%, 2%, 4% and 16%. The figure shows that the addition of Ag NPs does not change the 2θ values of the diffraction peaks of the DNTT thin films although the intensity of the peaks are relatively weaker after the addition of Ag NPs, especially at an Ag volume fraction of 16%. It suggests the introduction of Ag NPs at this volume fraction results in a more amorphous phase in the thin films, but in general, the lattice spacing in the DNTT is maintained. Nevertheless, a reduction in the thermal conductivity of the hybrid thin films when the Ag volume fraction increases may result from both an increased amorphous phase in the DNTT and boundary scattering at the Ag-DNTT interface. Both effects will be considered in the thermal conductivity modeling.

## Discussion

To simulate the thermal boundary scattering effect and evaluate the thermal conductivity of the hybrid DNTT thin films with a small Ag volume fraction, we applied an effective medium approximation (EMA). In the EMA calculation, we considered the size effect of Ag NPs and the electron-phonon coupling effect of Ag by combining the EMA model found in Minnich and Chen^33^ and that in Ordonez-Miranda *et al*.^34^ (see Supplementary Information for details). Cross-sectional TEM images in Fig. 4(b) indicate that the thin film is most aptly described as a three-layer structure where the nanocomposite layer (described by EMA) is sandwiched between two DNTT layers. To determine the volume fraction (higher than the effective volume fraction inclusive of all three layers) in this concentrated nanocomposite region of Ag for the EMA model, we assumed that spherical NPs were evenly distributed in the in-plane direction of the DNTT thin films and there was just one NP in each simple cube of DNTT. The NP diameter and cube length of a simple cube of DNTT were determined by the Ag NP distribution in the top view of the SEM images at different Ag volume fractions (see Supplementary Information for details). Figure S2(b) in Supplementary Information shows the diameter distribution and the average diameter of Ag NPs when the Ag volume fraction increases from 2% to 16%. However, when the Ag volume fraction is above 20%, according to SEM images of Fig. 4(a), Ag NPs are agglomerating to form a more continuous island structure, and the EMA model cannot be applied. Although the actual Ag NPs’ dispersion may vary with position, we assume a uniform distribution of Ag NPs in our EMA model which does not cause a significant deviation in the thermal conductivity (see Fig. S3 in Supplementary Information).

Since the total thickness of the film is kept constant at 50 nm, the nanocomposite layer becomes thicker and dominates the overall thermal conductivity of the hybrid films as Ag volume fraction increases. Two parameters in the EMA model were used to fit the *k*_*film*_ data: (i) the thermal boundary resistance between Ag and DNTT, and (ii) the modified thermal conductivity of the DNTT in the nanocomposite layer induced by crystallinity modification (see Supplementary Information for the details of EMA model). We assume that the thermal boundary resistance and DNTT thermal conductivity in the nanocomposite layer are constant over the range of Ag volume fractions in the EMA model. The averaged Ag NP diameter and the corresponding DNTT cube length are shown in Fig. 5(b). By comparing the EMA calculation and the experimental results, we find that the modified thermal conductivity of the DNTT in the nanocomposite layer is 0.31 ± 0.03 W/m-K and the TBR between Ag NPs and DNTT is 1.14 ± 0.98 × 10^−7^ m^2^-K/W (ranging between 1.60 × 10^−8^ m^2^-K/W and 2.12 × 10^−7^ m^2^-K/W). With these parameters, favorable comparison between the EMA model and the experimental *k*_*film*_ data is shown in Fig. 5(c). The evaluated TBR range is one to two orders of magnitude higher than the TBR between silicon and common metals such as Au or Al^35^,^36^,^37^, but it is comparable to that reported for the Ag-CuPc interface based on both molecular dynamics simulations and experiments^13^,^14^,^15^,^38^. This suggests the efficiency of heat dissipation between the metal and organic semiconductors are very different from the inorganic counterparts. The TBR value reported here is obtained under a moderate growth rate of materials (0.5 Å/s for DNTT, 2 Å/s for Ag) under room temperature. We believe that the measured TBR values are strongly dependent on the growth conditions of the metal-organic hybrid thin films as well as the nature of the metal-organic interface. For example, nanocrystal arrays are a related material where organic ligands are covalently bound to inorganic nanocrystals. In this case the surface chemistry between the ligands and core plays a major role, and the TBR is more than an order of magnitude smaller^39^,^40^. This vast variability controls thermal transport in organic-inorganic hybrid materials, and motivates more detailed investigations to create a universal understanding of TBR at hybrid interfaces.

To further validate the evaluated thermal boundary resistance, we applied finite element method (FEM) to simulate the thermal conductivity of the hybrid organic thin films. In the FEM simulation, we assumed the total thin film included three layers namely top DNTT layer, Ag-DNTT hybrid layer and bottom DNTT layer. When Ag volume fraction is low, Ag-DNTT hybrid layer consists of random distributed Ag NPs; while when Ag volume fraction is high, Ag forms a more continuous island structure (see Supplementary Information for simulation details). The simulated thermal conductivity of the whole thin film as a function of Ag volume fraction is plotted in Fig. 5(c). When the Ag volume fraction is relatively low in the hybrid thin film, the simulated results from FEM match well with the experimental and EMA results. However, when the Ag volume fraction is high, the simulated results deviate from the experimental value. We attribute this to the aggregation of the Ag NPs and formation of a more continuous Ag layer that outweigh the detrimental TBR effect. Other possible factors such as a reduction of the TBR of Ag-DNTT interface or an enhancement of the DNTT thermal conductivity in Ag-DNTT hybrid layer may increase the thermal conductivity of the hybrid thin film when the Ag volume fraction is high. As an example we show that reducing the TBR of Ag-DNTT interface by an order of magnitude to 1.60 × 10^−8^ m^2^-K/W describes the observed thermal conductivity at the highest Ag volume fraction. Nonetheless, truly discriminating between these factors would be difficult and is beyond the scope of this study.

In summary, we have demonstrated a general approach to modify the thermal conductivity of small molecule organic semiconductor thin films through the addition of metal NPs. The differential 3-ω method is utilized to measure the *k*_*film*_ of the Ag-DNTT hybrid thin films. We find that the grain size of the DNTT increases with increasing deposition temperature from 24 °C to 80 °C, but it does not affect the *k*_*film*_. The *k*_*film*_ of the pure DNTT thin film deposited at 24 °C is just 0.45 ± 0.06 W/m-K. After adding an Ag NP layer between the two pure DNTT layers, *k*_*film*_ initially decreases and then increases when the Ag volume fraction increases from 0% to 32%. By applying a combined EMA model taken from two previous studies to determine the thermal conductivity, and comparing the calculated result with the experimental result, the TBR of the Ag-DNTT interface is extracted as 1.14 ± 0.98 × 10^−7^ m^2^-K/W when the Ag volume fraction is low. We further applied FEM to simulate the thermal conductivity of the hybrid thin film which matches well with the experimental results. Due to the low thermal conductivity of organic semiconductors and inferior thermal transport of metal-organic semiconductors, thermal management in organic electronics is critical to the device performance especially for scaled-up large area applications. Our findings that the thermal conductivity of organic semiconductor is influenced by the addition of metal NPs bears on thermal management and thermoelectric applications for organic semiconductors.

## Methods

### Sample fabrication

**Figure 1:**
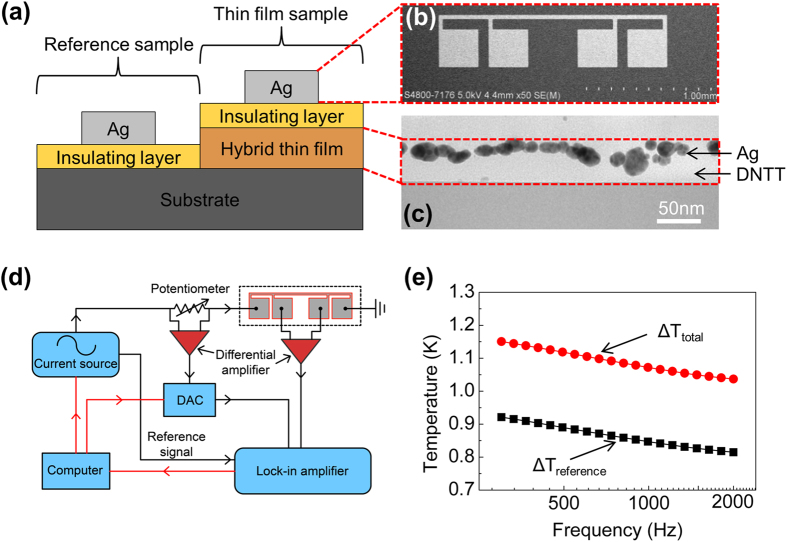
(**a**) Structure of the thin film and the reference samples. (**b**) A top-view SEM image of an Ag 3-ω heater/thermometer. (**c**) A cross-sectional TEM image of a hybrid thin film with an Ag volume fraction of 16%. (**d**) Schematic of the 3-ω measurement setup. (**e**) Typical temperature vs. 1-ω frequency profiles for a thin film sample (red dots) and a reference sample (black squares).

**Figure 2:**
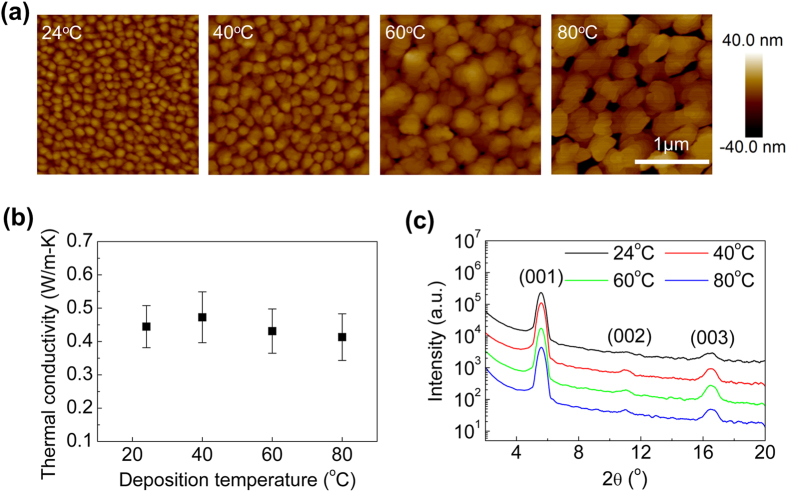
(**a**) AFM images of surface morphology of DNTT thin films at different deposition temperatures, 24 °C, 40 °C, 60 °C and 80 °C. (**b**) Thermal conductivity of 50 nm thick DNTT films made with different deposition temperatures. (**c**) Out-of-plane XRD spectra of 50 nm thick DNTT films made with different deposition temperatures.

**Figure 3:**
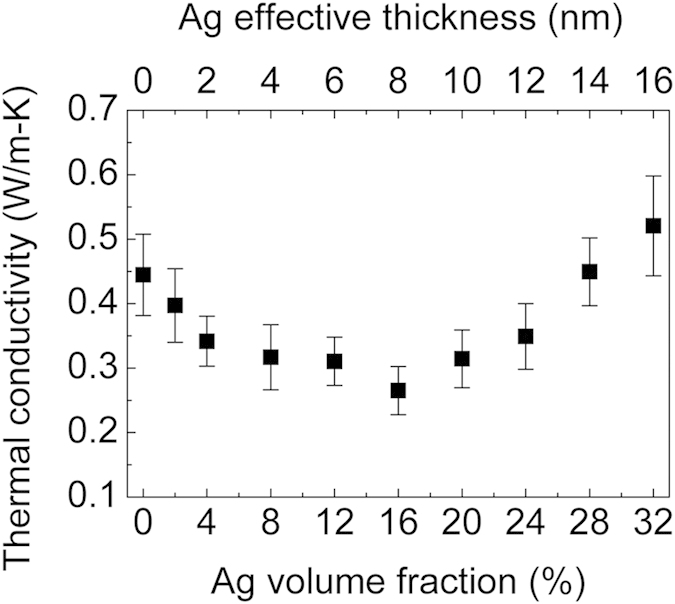
Thermal conductivity of Ag-DNTT hybrid thin films deposited at 24 °C with Ag volume fraction ranging from 0% to 32%. The total thickness of the thin film is 50 nm and the effective thickness of the Ag layer is varied from 0 to 16 nm.

**Figure 4:**
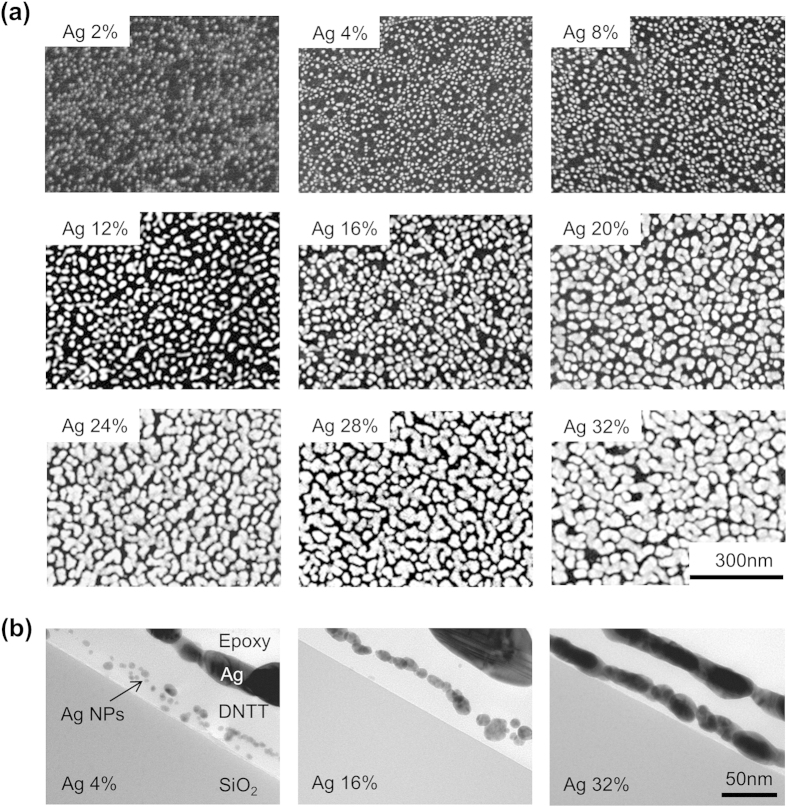
(**a**) SEM images of Ag layer (bright area) on the bottom DNTT layer (dark area) deposited at 24 °C with Ag volume fraction ranging from 2% to 32%. (**b**) Cross-sectional TEM images of thin films deposited at 24 °C with Ag volume fractions of 4%, 16% and 32%.

**Figure 5:**
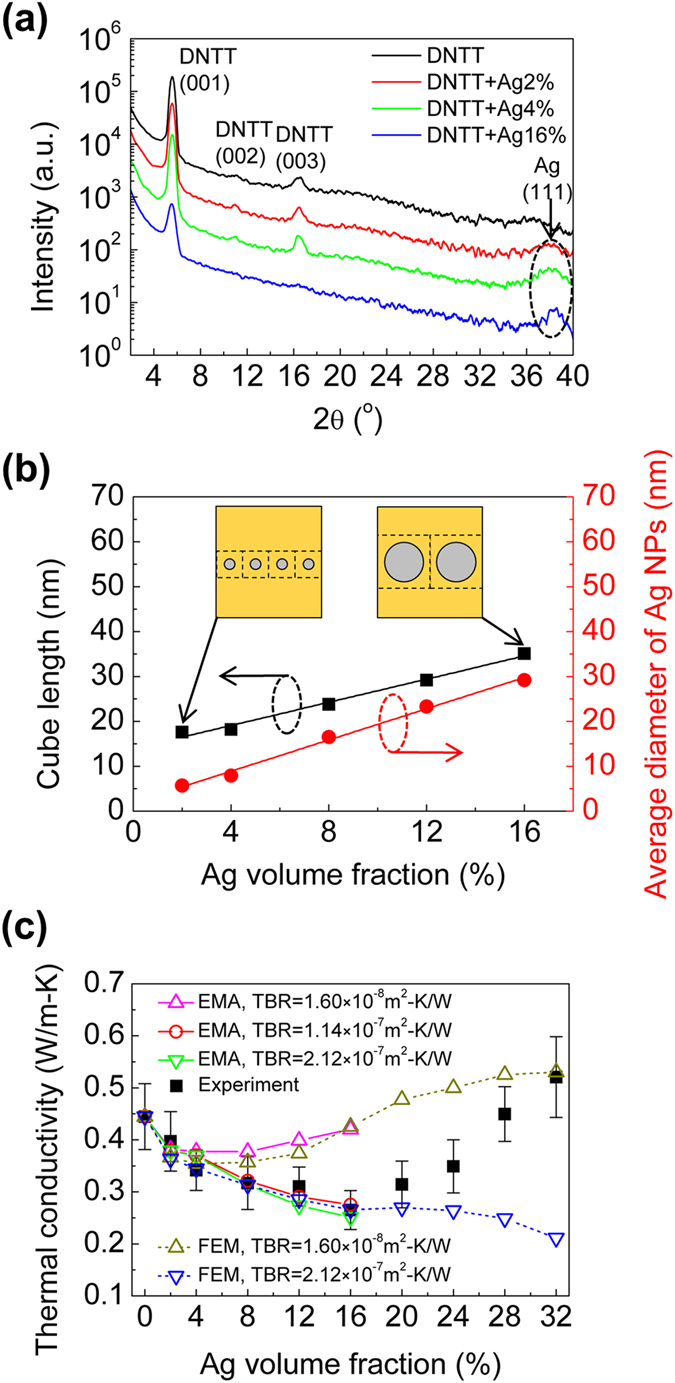
(**a**) Out-of-plane XRD spectra of Ag-DNTT hybrid thin films deposited at 24 °C with Ag volume fractions of 0%, 2%, 4% and 16%. (**b**) Cube length of the DNTT simple cube (black squares) and average diameter of Ag NPs (red dots) in EMA model with Ag volume fraction ranging from 2% to 16%. (**c**) Thermal conductivity of Ag-DNTT hybrid thin films with Ag volume fraction ranging from 0% to 32%. Black solid square symbols represent the experimental results, solid lines with open symbols represent EMA calculated results, and dashed lines with open symbols represent FEM simulated results. In EMA calculation and FEM simulation, the modified thermal conductivity of DNTT in nanocomposite or Ag-DNTT hybrid layer is 0.31 W/m-K.

The schematic diagram of the sample structures is shown in Fig. 1(a). A heavily doped silicon (Si) wafer (University Wafer, ~500 μm thick) with thermally-grown silicon dioxide (SiO_2_; 300 nm in thickness) was used as the substrate. DNTT (Lumtec, sublimed grade 99%) and Ag (Kurt J. Lesker, 99.99% pure) were deposited by using a thermal evaporator with a base pressure of 5 × 10^−7^ torr. Prior to the deposition, the substrate was successively cleaned by using deionized water, acetone, and iso-propanol, followed by 15-min oxygen plasma treatment. The Ag-DNTT hybrid thin films of interest, with different Ag volume fractions varying from 0% to 32%, were deposited layer-by-layer as reported in earlier work^2^,^22^. As shown in Fig. 1(c), a discontinuous Ag NP layer is sandwiched between two DNTT layers. The *in-situ* substrate temperature during the deposition process was controlled by a heater. The total thickness of the thin film of interest was kept at 50 nm. The Ag heater/thermometer for the 3-ω measurement was deposited by thermal evaporation with a thickness of 100 nm and patterned by a shadow mask to a width of 29 μm. An SEM (Hitachi S4800 field emission scanning electron microscope) image of a heater is shown in Fig. 1(b). The uncertainties in thickness and width are less than 10%, as confirmed by an atomic force microscopy (AFM, Bruker MultiMode 8) and a scanning electron microscope (SEM, Hitachi S4800). The crystallinity of the thin films was investigated with a XRD (Rigaku SmartLab). The cross-sectional TEM images were performed with a transmission electron microscope (TEM, PHILIPS CM100). To avoid the parasitic leakage current in the device, a 30 nm insulation layer of pure DNTT was deposited on top of the thin film. Here, we applied the differential 3-ω method^25^, and a reference sample without the thin film of interest was employed as the control (Fig. 1(a)). On each substrate, 20 thin film devices and 20 reference devices were patterned by the shadow mask.

### 3-ω measurement

The schematic diagram of the differential 3-ω method is shown in Fig. 1(d). An AC current with a frequency of 1-ω is driven through two outer metal pads, which gives rise to joule heating at a frequency of 2-ω. The wire dissipates heat into the sample and hence its temperature rise is determined by thermal transport properties of the sample. Based on the temperature coefficient of resistance (TCR), the wire’s resistance will be modulated at a frequency of 2-ω. Because the voltage between two inner metal pads results from the product of 1-ω current and 2-ω resistance signals, a 3-ω voltage signal can be used to extract the temperature increase (Δ*T*) as follows:


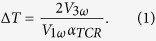


where, *V*_*1ω*_, *V*_*3ω*_, *α*_*TCR*_ are 1-ω voltage, 3-ω voltage, and TCR of the metal heater. 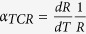, where *R* is the resistance of the metal heater. In our differential 3-ω measurement setup, we derived the temperature increase of the thin film of interest (Δ*T*_*film*_) based on the temperature increases of the thin film (Δ*T*_*total*_) and reference sample (Δ*T*_*reference*_). So the thermal conductivity of the thin film of interest is given by^25^:


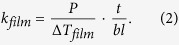


where, *P*,*t*, *b* and *l* are the input power, the thickness of the thin film, and the width and length of the metal heater. Because the width of the heater is orders of magnitudes higher than the thickness of the thin film, we neglect the in-plane heat spreading effect in the hybrid thin film^41^. The uncertainty of the thermal conductivity considers standard deviations of measured 3-ω voltage of different devices on the thin film sample and reference sample, uncertainties of TCR, width and length of the metal heater, and uncertainty of thickness of the thin film.

We used an AC current source (Keithley 6221) to drive the 1-ω current and supply a synchronous reference signal for the lock-in amplifier (Stanford Research System, SR850). Then, we used the lock-in amplifier to measure the 3-ω voltage signal. Two typical temperature vs. frequency profiles-one of the thin film sample (Ag volume fraction of 0%) and one of the reference sample-are shown in Fig. 1(e). The temperature difference between the thin films (Δ*T*_*total*_) and references (Δ*T*_*reference*_) is utilized to calculate the thermal conductivity based on equation (2)^25^,^41^. It is noteworthy that because we used DNTT as the insulating layer, the thermal boundary resistance between the insulating layer and the thin film of interest is zero. To confirm the electrical insulation of DNTT, we cut off the metal wire, measure the resistance of different pads and open circuit is obtained. All of the 3-ω measurements were performed in a vacuum cryostat (Janis ST-500) with a base pressure below 5 × 10^−4^ torr using four micro-manipulated probes.

## Additional Information

**How to cite this article**: Wang, X. *et al.* Modifying the thermal conductivity of small molecule organic semiconductor thin films with metal nanoparticles. *Sci. Rep.* **5**, 16095; doi: 10.1038/srep16095 (2015).

## Supplementary Material

Supplementary Information
