# Supplementary material for: Modifying the thermal conductivity of small molecule organic semiconductor thin films with metal nanoparticles
Source: Sci Rep. 2015 Nov 4;5:16095. doi: 10.1038/srep16095 (PMC4632039; doi:10.1038/srep16095)
Supplement: Supplementary Information [file srep16095-s1.docx]

*Supplementary information for*

**Modifying the thermal conductivity of small molecule organic semiconductor thin films with metal nanoparticles**

Xinyu Wang^1^, Kevin D. Parrish^2^, Jonathan A. Malen^2^ and Paddy K. L. Chan^1*^

*^1^**Department of Mechanical Engineering, The University of Hong Kong, Hong Kong*

^2^*Department of Mechanical Engineering, Carnegie Mellon University, Pittsburgh, Pennsylvania, USA*

**Correspondence and requests for materials should be addressed to P.K.L.C (pklc@hku.hk)*

1. **Effective medium approximation**

Effective medium approximation (EMA) is widely utilized to theoretically determine the effective thermal conductivity of nanocomposite materials^1-4^. Nan et al. developed a general model to determine the thermal conductivity of arbitrary nanocomposites by considering the effect of TBR^2^. Minnich et al. further improved the model developed by Nan et al., where the phonon mean free path (MFP) was modified with NP size^3^. Ordonez-Miranda et al. used a two-temperature model and EMA to propose an equation to determine the effective thermal conductivity of metal-nonmetal nanocomposites^4^. Here, we applied EMA to calculate the thermal conductivity of hybrid thin films with a small Ag volume fraction. By fitting the experimental result with the calculated result of thermal conductivity of hybrid thin films (*k_film_*), the TBR of the Ag-DNTT interface could be extracted.

In our EMA calculation, the whole hybrid thin film could be considered as a three-layer structure with an Ag-DNTT nanocomposite layer sandwiched between two DNTT layers, as shown in Fig. S1. For the nanocomposite layer, we assumed that the NPs were spherical and there was just one NP in each simple cube of DNTT. So the thermal conductivity of the hybrid thin films is calculated by using the following equations:

$\frac{L_{total}}{k_{\text{film}}}=\frac{L_{total}-l}{k_{\text{DNTT}}}+\frac{l}{k_{\text{composite}}}$ (S1)

where, *k_DNTT_*, and *k_composite_* are the experimentally measured thermal conductivity of pure DNTT film, and the thermal conductivity of the nanocomposite layer (which takes into consideration the thermal boundary resistance between the metal and the organic semiconductor) respectively; *L_total_* and *l* are the total thickness of the hybrid thin film and the cube length of the simple cube of the DNTT, respectively.


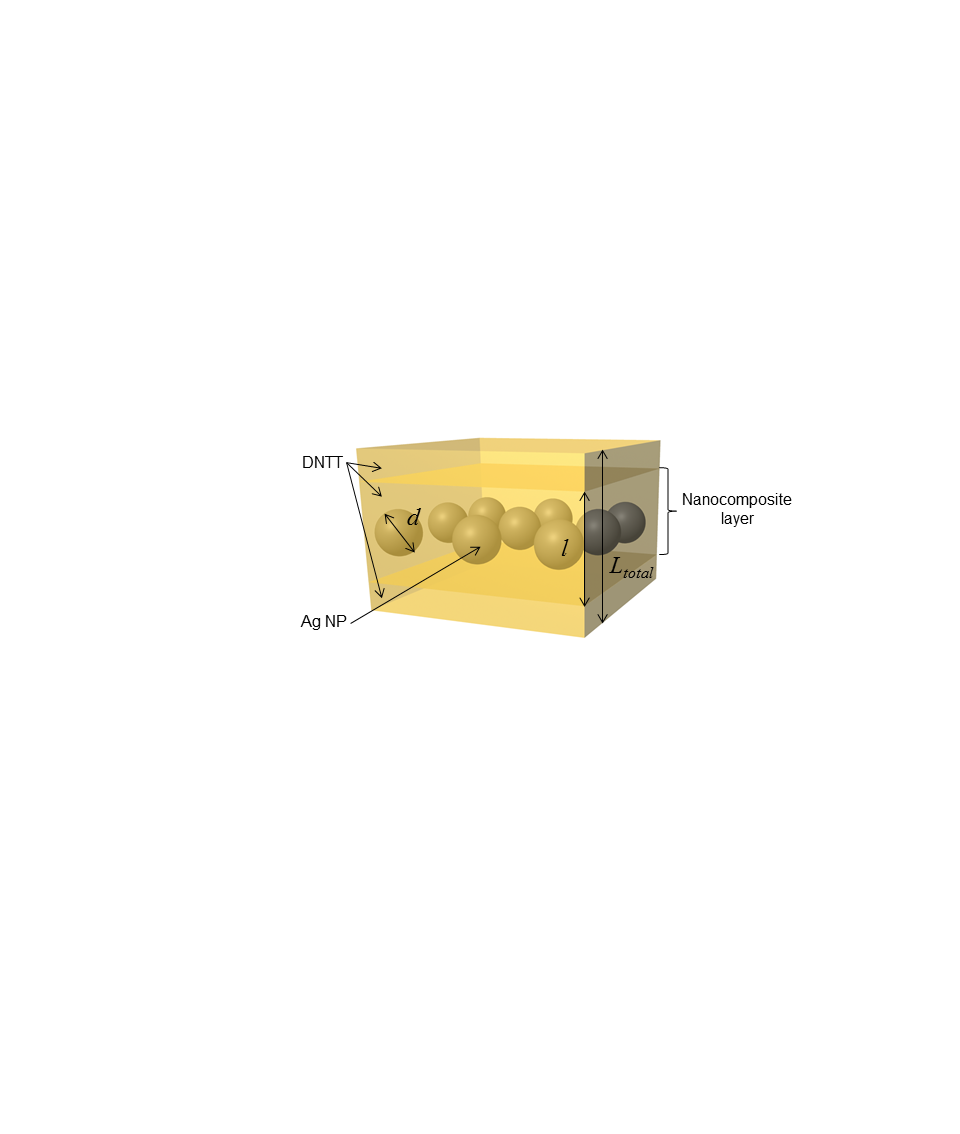


Figure S1| Structure of the hybrid thin film in EMA model

To calculate the thermal conductivity of the nanocomposite layer, we combined the models from Minnich and Chen^3^ and Ordonez-Miranda et al.^4^ into our calculation. According to the former, the effective electron and phonon MFPs of Ag NPs and effective phonon MFP of DNTT can be modified by using Matthiessen’s rule, respectively.

$\frac{1}{\lambda_{Ag,e,eff}}=\frac{1}{\lambda_{Ag,e,bulk}}+\frac{1}{d}$ (S2)

$\frac{1}{\lambda_{Ag,p,eff}}=\frac{1}{\lambda_{Ag,p,bulk}}+\frac{1}{d}$ (S3)

$\frac{1}{\lambda_{DNTT,eff}}=\frac{1}{\lambda_{DNTT,composite}}+\frac{\pi d^{2}}{4l^{3}}$ (S4)

where, *λ* is the MFP and *d* is the diameter of the Ag NP. Subscripts *e*, *p*, and *composite* stand for electron, phonon, and the nanocomposite layer. In our calculation, *λ_Ag,p,bulk_* is 9.5 nm based on the literature^5^. *λ_Ag,e,bulk_* is 61.5 nm and *λ_DNTT,composite_* is 3.1 nm, which are extracted by fitting the experimental thermal conductivity value with the lattice Boltzmann method that we have previously reported^6^.

Based on the kinetic theory of thermal conductivity, the effective thermal conductivities of Ag and DNTT are related to the corresponding mean free paths (Eq. S2-S4) as follows:

$\begin{matrix} k_{Ag,eff}=k_{Ag,e,eff}+k_{Ag,p,eff}=\frac{1}{3}C_{Ag,e}v_{Ag,e}\lambda_{Ag,e,eff}+\frac{1}{3}C_{Ag,p}v_{Ag,p}\lambda_{Ag,p,eff} \\ \text{ =}k_{Ag,e,bulk}\frac{\lambda_{Ag,e,eff}}{\lambda_{Ag,e,bulk}}+k_{Ag,p,bulk}\frac{\lambda_{Ag,p,eff}}{\lambda_{Ag,p,bulk}} \\ \text{ } \end{matrix}$ (S5)

$k_{DNTT,eff}=\frac{1}{3}C_{DNTT}v_{DNTT}\lambda_{DNTT,eff}=k_{DNTT\text{,composite}}\frac{\lambda_{DNTT,eff}}{\lambda_{DNTT,composite}}$ (S6)

where, *C* is the volumetric specific heat and *v* is the group velocity.

By combining the size effect and the model by Ordonez-Miranda et al.^4^, the thermal conductivity of the nanocomposite layer, last term in Eq. S1, is given as

$k_{composite}=k_{DNTT,eff}\frac{k_{Ag,eff}(1+2r)+2\chi k_{DNTT,eff}+2\left[k_{Ag,eff}(1-r)-\chi k_{DNTT,eff}\right]f}{k_{Ag,eff}(1+2r)+2\chi k_{DNTT,eff}-\left[k_{Ag,eff}(1-r)-\chi k_{DNTT,eff}\right]f}$ (S7)

where,

$\chi=1+\frac{k_{Ag,e,eff}}{k_{Ag,p,eff}}\frac{2\beta}{d}\frac{i_{1}\left(d/(2\beta)\right)}{i'_{1}\left(d/(2\beta)\right)}$ (S8a)

$\beta=\sqrt{(1/G)(k_{Ag,e,eff}k_{Ag,p.eff}/(k_{Ag,e,eff}+k_{Ag,p,eff}))}$ (S8b)

$r=\frac{2\cdot k_{DNTT,eff}\cdot R_{TBR}}{d}$ (S8c)

where, *G* is the Ag electron-phonon coupling factor, where we use the results from the literature^7,8^, 3.5×10^16^ W/(m^3^-K). *i*_1_( ) and *i*_1_'( ) are the modified spherical Bessel function of the first kind and order one and its derivative respectively. *f* represents the Ag volume fraction in one DNTT cube and *R_TBR_* is the TBR of the Ag-DNTT interface.

The diameter of the NP and cube length in the EMA calculation are determined by the Ag NP distribution in the SEM images at different Ag volume fractions. We assume spherical NPs are planarly distributed in the in-plane direction of the DNTT thin films (planar distribution). As shown in Fig. S2(a), we first convert the original SEM image into a binary image and as such, are able to calculate the Ag area ratio to DNTT and the number of Ag NPs in the binary image by using MATLAB software. The diameter distribution of the Ag NPs can be obtained when Ag NP volume fraction increases from 2% to 16% as shown in Fig. S2(b). Finally, to simplify the EMA model, we assume that the diameter of Ag NP is uniform, the cube length of the simple cube of the DNTT and average diameter of the Ag NPs can be evaluated by use of the Ag area ratio and NP number as shown in Fig. 5(b).

After obtaining the diameter of NP and cube length of the Ag-DNTT composite, we used Eq. (S7) to calculate the thermal conductivity of composite thin film (*k_composite_*) and apply it into Eq. (S1) to evaluate *k_film_* which can be fitted with the experimental result obtained by 3-ω method. In Eq. (S7), we additionally considered the change in the thermal conductivity of the DNTT in the nanocomposite layer induced by the crystallinity modification of Ag in the calculation. By fitting the experimental results with the calculated values of *k_film_* from Eq. (S1), the modified thermal conductivity of the DNTT in the nanocomposite layer (*k_DNTT,composite_*) and TBR between Ag and DNTT in Eq. (S7) can be extracted. We find the modified thermal conductivity of the DNTT in the nanocomposite layer (*k_DNTT,composite_*) is 0.31±0.03 W/m-K and TBR between Ag and DNTT is 1.14±0.98×10^-7^ m^2^-K/W (bounding between 1.60×10-8 m2-K/W and 2.12×10-7 m2-K/W).

To verify our EMA model assuming Ag NPs are dispersed by the planar distribution in DNTT, we calculated the thermal conductivity by EMA by assuming 3D Ag NP dispersion with different dispersion heights (from 10 nm to 50 nm) in DNTT when Ag volume fractions are 2% and 4%. From Fig. S3, we can find that even though cube length increases with dispersion height increasing from 10 nm to 50 nm, the thermal conductivity calculated by EMA varies very limited for Ag volume fractions of 2% and 4%. Therefore, it is reasonable to assume a planar distribution of Ag NPs in EMA. In addition, it can be noted that when Ag volume fraction is high, Ag NPs nearly distribute in one planar layer as shown in TEM images of Fig. 4(b). Hence for high Ag volume fraction, in EMA model we do not need to consider 3D Ag NP dispersion in DNTT.


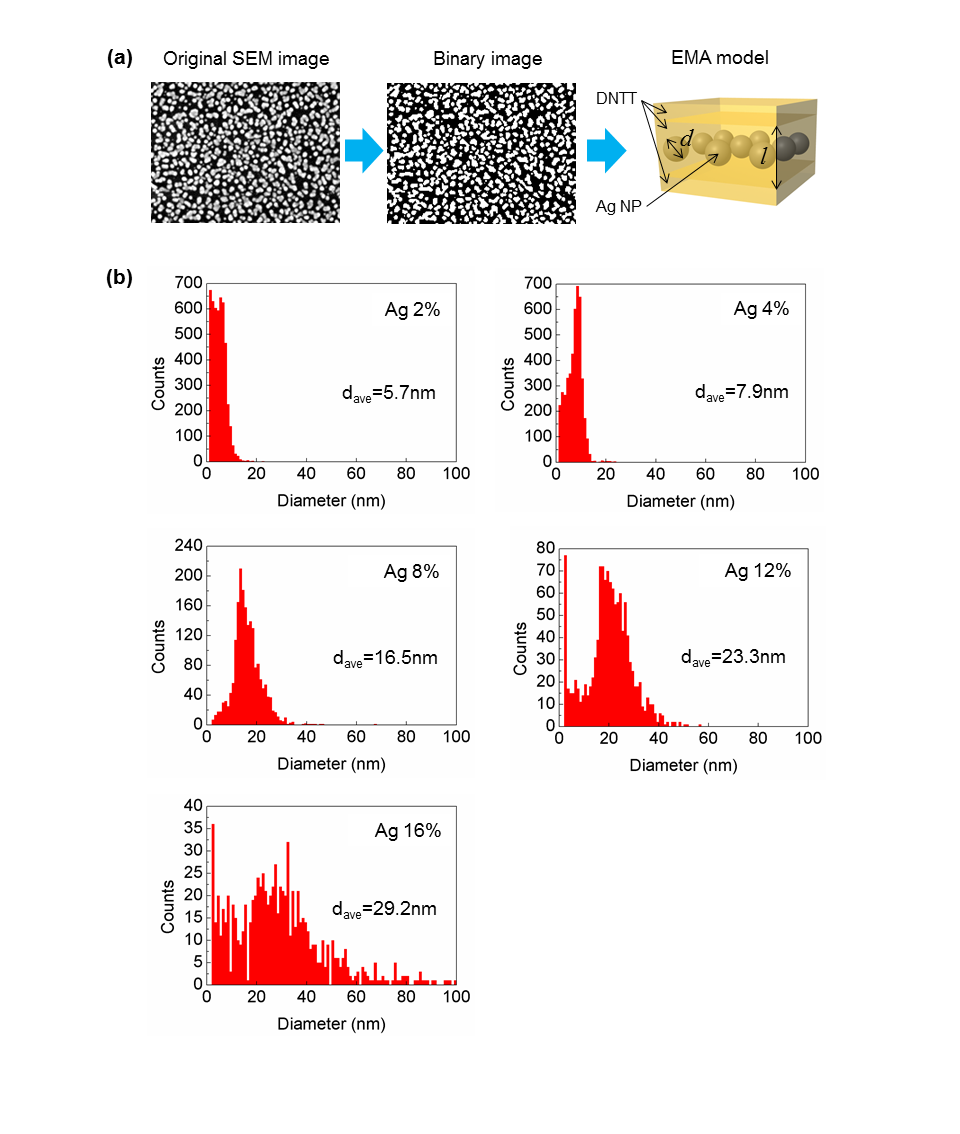


Figure S2| (a) Process of determining cube length of the DNTT simple cube (*l*) and average diameter of Ag NPs (*d*). (b) Ag NP diameter distribution with Ag volume fraction ranging from 2% to 16%.


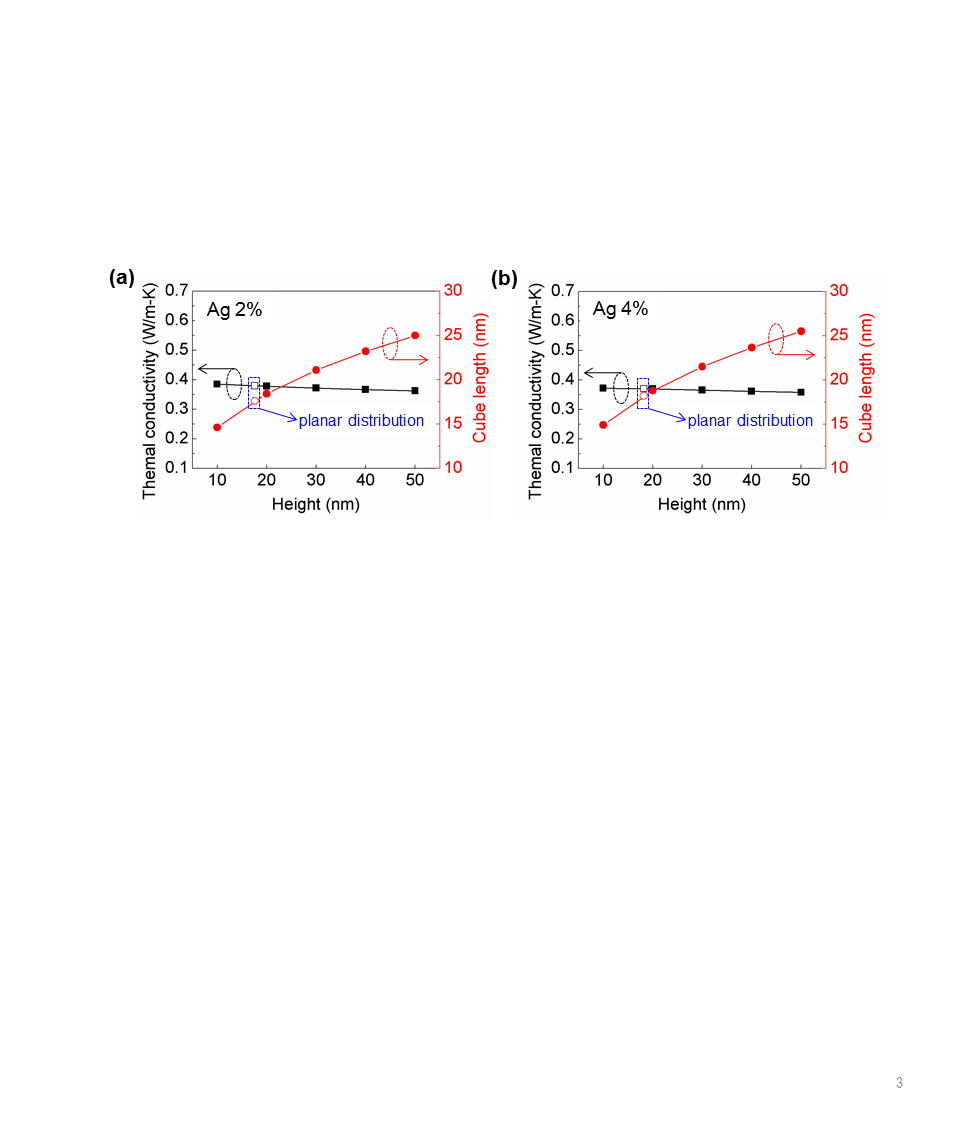


Figure S3| Thermal conductivity and cube length with Ag NP dispersion height in DNTT ranging from 10 nm to 50 nm for Ag volume fractions of (a) 2% and (b) 4%. Open symbols represent the assumption of the planar distribution. In the calculation, the modified thermal conductivity of DNTT in nanocomposite is 0.31 W/m-K and TBR between Ag and DNTT is 1.14×10^-7^ m^2^-K/W.

1. **Finite element simulation**

Finite element method (FEM) was applied to simulate the thermal conductivity by using commercial software (COMSOL multiphysics)^9^. In the FEM simulation, we assumed the total thin film included the top DNTT layer, Ag-DNTT hybrid layer and bottom DNTT layer. When Ag volume fraction is low, Ag form is assumed as random distributed Ag NPs. We used a 3D drawing softeware (3ds Max) to develop the random distribution of Ag NPs based on the particle diameter distribution in Fig. S2 (b) and these nanoparticles are placed in the hybrid layer as shown in Fig. S4. When Ag volume fraction is high, Ag structure shows a more continuous island. In the simulation model (Fig. S4), thermal conductivity of top and bottom DNTT layer is 0.45 W/(m-K), and thermal conductivity of DNTT with crystallinity modification is 0.31 W/(m-K), which is obtained by EMA fitting. To consider the thermal boundary resistance (TBR), we set the boundary condition between Ag and DNTT as “thin thermally resistive layer” in which thermal resistance used the TBR we have fitted from EMA, ranging between 1.60×10^-8^ m^2^-K/W and 2.12×10^-7^ m^2^-K/W. From Fig. 1(e), we can observe that the temperature difference across the thin film is around 0.2 K in the experiment. Hence we applied 0.2 K temperature difference between top surface and bottom surface of the whole thin film in the simulation model. By calculating the heat flux across the thin film, the thermal conductivity of the thin film can be evaluated.


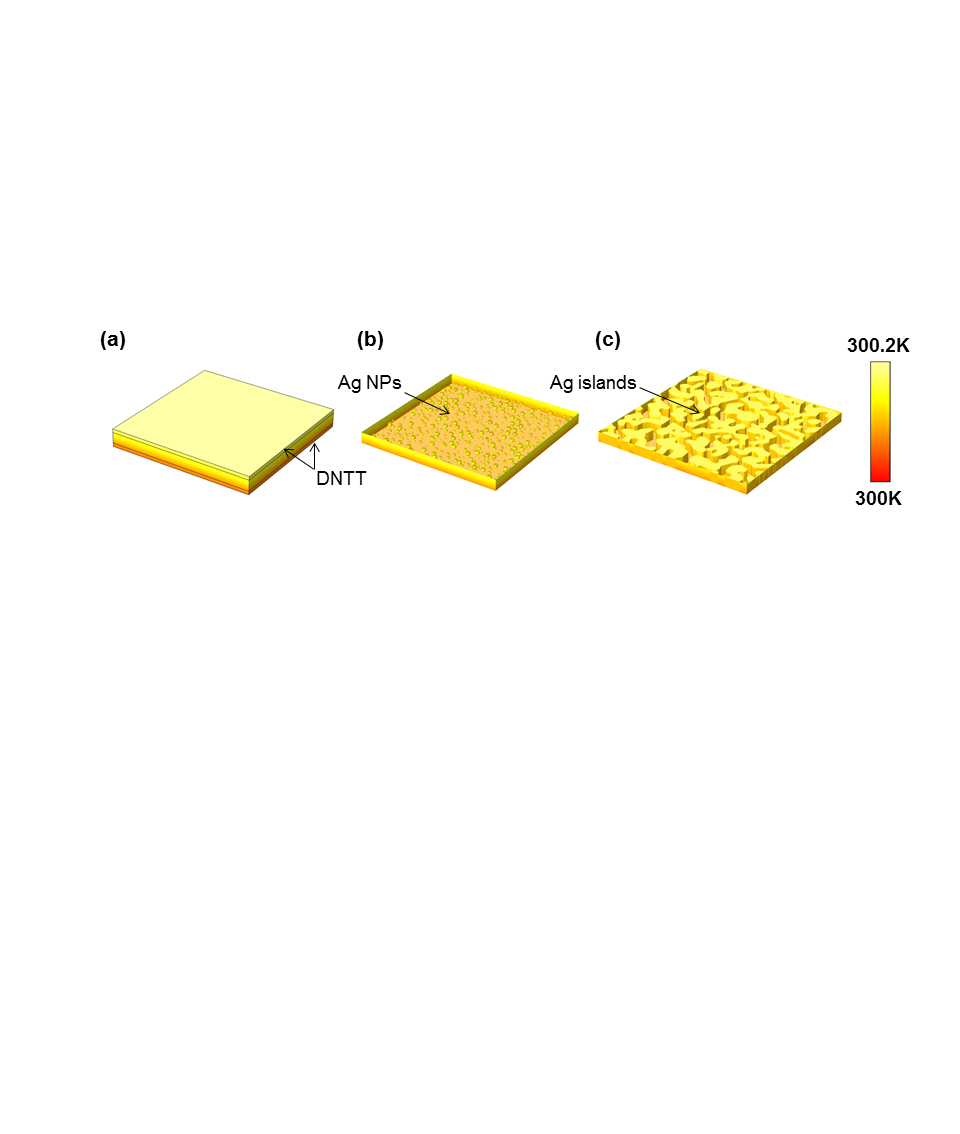


Figure S4| Simulation model in finite element method. (a) Temperature distribution of 3 layers of thin film. (b) Temperature distribution of Ag NPs. (c) Temperature distribution of Ag islands. (DNTT in the top, bottom and middle hybrid layers are set as transparent)

1. **Uncertainty analysis**

The uncertainty of the thermal conductivity considers standard deviations of measured 3-ω voltage of different devices on the thin film sample and reference sample, uncertainties of TCR, width and length of the metal heater, and uncertainty of thickness of the thin film. The detailed uncertainty of the thermal conductivity, *Δk*, is calculated as follows^10^:

$\Delta k=\sqrt{\sum\left(\frac{\partial k_{i}}{\partial\gamma_{i}}\Delta\gamma_{i}\right)^{2}}$ (S9)

where, *γ_i_*, represents the 3-ω voltage, thickness of the thin film, and TCR, width and length of the metal heater. The nominal values and uncertainties of TCR, film thickness, heater width and length are as shown in Table S1. The uncertainties in thickness, width and length are confirmed by the atomic force microscopy (AFM, Bruker, MultiMode 8) and scanning electron microscope (SEM, Hitachi S4800). To determine the TCR of the heater, we measure the heater resistance at different temperatures from 290 K to 310 K as shown in Fig. S5. The TCR of heater resistance at 300 K is 2.11×10^-3^ K^-1^. Based on the uncertainties of these parameters and standard deviations of measured 3-ω voltage of different devices, the uncertainty of thermal conductivity ranges from 11% to 17%. The error bar of thermal conductivity has shown in Fig. 3 of the main text.


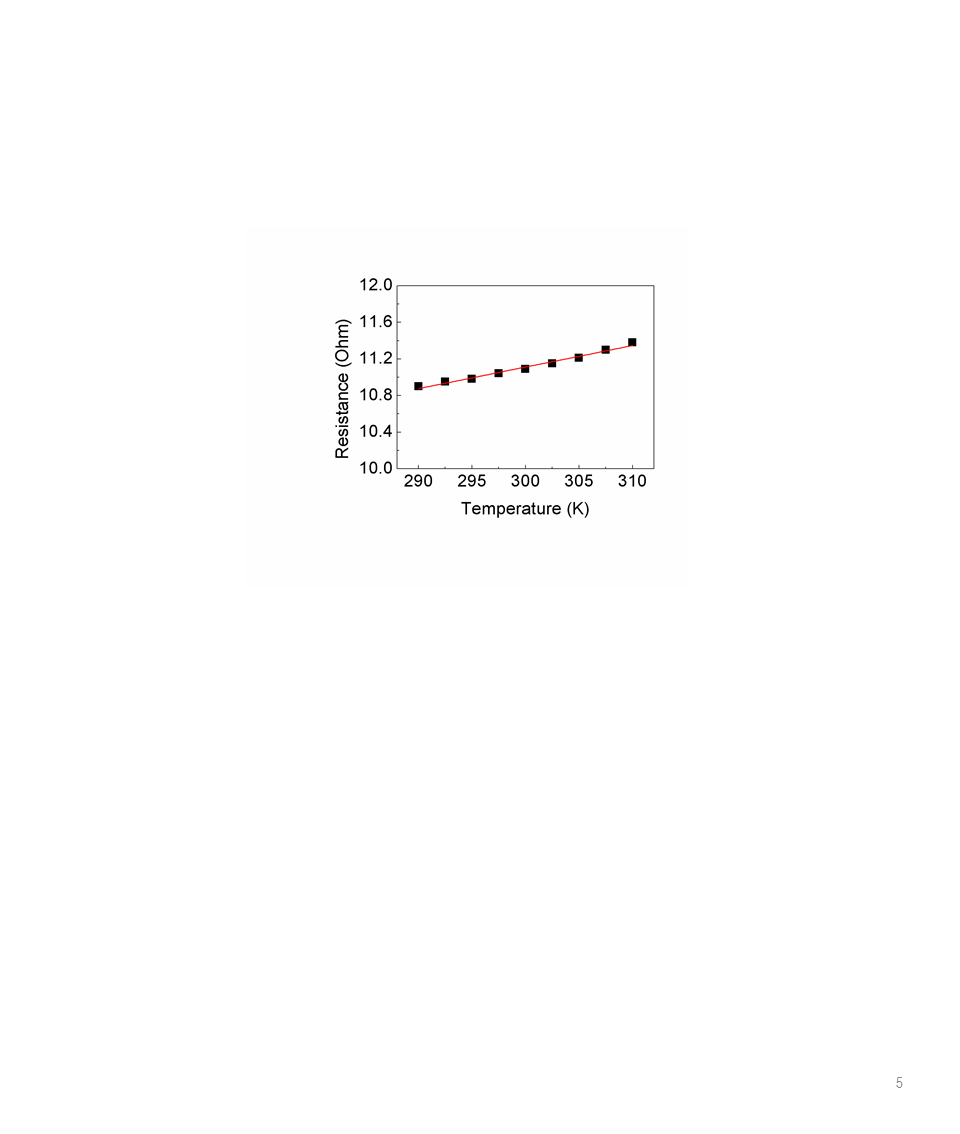


Figure S5| Heater resistance at different temperatures from 290 K to 310 K.

Table S1 Nominal values and uncertainties of TCR, film thickness, heater width and length

| *γ_i_*, | Nominal value | Uncertainty |
| --- | --- | --- |
| Heater TCR (K^-1^) | 2.11×10^-3^ | ±3.85×10^-5^ |
| Film thickness (nm) | 50 | ±5 |
| Heater width (μm) | 29 | ±1 |
| Heater length (μm) | 1000 | ±10 |

**Reference**

1 Torquato, S. & Rintoul, M. D. Effect of the interface on the properties of composite media. *Phys. Rev. Lett.* **75**, 4067-4070 (1995).

2 Nan, C. W., Birringer, R., Clarke, D. R. & Gleiter, H. Effective thermal conductivity of particulate composites with interfacial thermal resistance. *J. Appl. Phys.* **81**, 6692-6699 (1997).

3 Minnich, A. & Chen,G. Modified effective medium formulation for the thermal conductivity of nanocomposites. *Appl. Phys. Lett.* **91**, 073105 (2007).

4 Ordonez-Miranda, J., Yang, R. G. & Alvarado-Gil, J. J. A model for the effective thermal conductivity of metal-nonmetal particulate composites. *J. Appl. Phys.* **111**, 044319 (2012).

5 Heino, P. & Ristolainen, E. Thermal conduction at the nanoscale in some metals by MD. *Microelectron. J.* **34**, 773-777 (2003).

6 Wang, X. & Chan, P. K. L. in *ASME 2014 International Mechanical Engineering Congress and Exposition*. (Montreal, Quebec, Canada, 2014), vol. 8A: Heat Transfer and Thermal Engineering, pp. V08AT10A005.

7 Groeneveld, R. H. M., Sprik, R. & Lagendijk, A. Ultrafast relaxation of electrons probed by surface plasmons at a thin silver film. *Phys. Rev. Lett.* **64**, 784-787 (1990).

8 Groeneveld, R. H. M., Sprik, R. & Lagendijk, A. Femtosecond spectroscopy of electron-electron and electron-phonon energy relaxation in Ag and Au. *Phys. Rev. B* **51**, 11433-11445 (1995).

9 Jin, Y., Nola, S., Pipe, K. P. & Shtein, M. Improving thermoelectric efficiency in organic-metal nanocomposites via extra-low thermal boundary conductance. *J. Appl. Phys.* **114**, 194303 (2013).

10 Regner, K. T., Majumdar, S. & Malen, J. A. Instrumentation of broadband frequency domain thermoreflectance for measuring thermal conductivity accumulation functions. *Rev. Sci. Instrum.* **84**, 064901, (2013).
